# Lack of serological evidence of Middle East respiratory syndrome coronavirus infection in virus exposed camel abattoir workers in Nigeria, 2016

**DOI:** 10.2807/1560-7917.ES.2018.23.32.1800175

**Published:** 2018-08-09

**Authors:** Ray TY So, Ranawaka APM Perera, Jamiu O Oladipo, Daniel KW Chu, Sulyman A Kuranga, Kin-ho Chan, Eric HY Lau, Samuel MS Cheng, Leo LM Poon, Richard J Webby, Malik Peiris

**Affiliations:** 1 School of Public Health, The University of Hong Kong, Hong Kong Special Administrative Region, China; 2 These authors contributed equally to this work; 3 Faculty of Clinical Sciences, Department of Surgery, Old Jebba Road, University of Ilorin, Ilorin, Nigeria; 4 Department of Infectious Diseases, St. Jude Children’s Research Hospital, Memphis, United States

**Keywords:** MERS, coronavirus, serology, occupational exposure, human, camel, abattoir

## Abstract

Middle East respiratory syndrome coronavirus (MERS-CoV) is a zoonotic threat of global public health concern and dromedary camels are the source of zoonotic infection. Although MERS-CoV is enzootic in dromedaries in Africa as well as the Middle East, zoonotic disease has not been reported in Africa. **Methods**: In an abattoir in Kano, Nigeria, we tested nasal swabs from camels and investigated 261 humans with repeated occupational exposure to camels, many of whom also reported drinking fresh camel milk (n = 138) or urine (n = 94) or using camel urine for medicinal purposes (n = 96). **Results**: Weekly MERS-CoV RNA detection in January–February 2016 ranged from 0–8.4% of camels sampled. None of the abattoir workers with exposure to camels had evidence of neutralising antibody to MERS-CoV. **Conclusion**: There is a need for more studies to investigate whether or not zoonotic transmission of MERS-CoV does take place in Africa.

## Introduction

Middle East respiratory syndrome coronavirus (MERS-CoV) is an ongoing threat to global public health [1]. Serological and virological studies have shown evidence of MERS-CoV infection in camels in the Middle East, as well as in East, North and West Africa [2-5] and in Central Asia [6]. In spite of MERS-CoV being enzootic in camels in Africa, zoonotic MERS has not been reported from the African continent. Our recent genetic and phenotypic analysis of MERS-CoV from camels in West (Burkina Faso, Nigeria) Africa has shown that West African viruses were phylogenetically and phenotypically distinct from those associated with human disease in the Arabian Peninsula [7], raising the possibility that virus strain differences may be associated with differences in zoonotic potential.

Abattoir workers with exposure to infected camels are a high-risk group for MERS-CoV infection in the Arabian Peninsula [8]. However, there is a paucity of serological data on MERS-CoV infection in people occupationally exposed to camels in Africa, a knowledge gap identified as a priority research question at a Food and Agriculture Organization of the United Nations-World Organisation for Animal Health-World Health Organization (FAO-OIE-WHO) Global Technical Meeting on MERS in September 2017 [1]. A previous study in Egypt in 2013 showed no serologic evidence of MERS-CoV among 179 serum samples from humans working in two camel abattoirs [3]. None of 760 people with household exposure to seropositive camels in Kenya in 2013 had evidence of MERS-CoV antibody [9]. Another study in Kenya in 2013–14 of 1,122 people (not with necessarily high exposure to camels) found two sera with low and inconclusive levels of neutralising antibody to MERS-CoV [10]. It remains important to carry out more sero-epidemiological studies on humans with occupational exposure to infected camels to understand whether or not zoonotic transmission is taking place in Africa. We therefore investigated for serological evidence of MERS-CoV infection of humans occupationally exposed to infected dromedary camels in an abattoir in Kano, Nigeria.

## Methods

### Study sites and sample collection

Around 70 camels are slaughtered daily at the abattoir in Kano, Nigeria. We collected around 20 nasal swabs daily from 12 October to 2 December 2015 and from 11 January to 29 February 2016. Swab samples were placed in viral transport medium and stored at -80°C.

Abattoir workers with and without occupational exposure to camels were recruited for a serological study after obtaining informed consent, during April–November 2016. A questionnaire was administered to each participant to ascertain demographic information, type and duration of occupational exposure to camels or other livestock, practices such as consuming camel milk or use of camel urine for food or health purposes. Camel exposures in the abattoir were classified as ‘direct’ (exposure to live or freshly slaughtered camels or camel meat) or ‘indirect’ (no exposure to live camels or freshly slaughtered camels or meat; exposure only being to cooked meat or dried bones etc. as further described in the Table). Duration of exposure to camels in the abattoir was categorised as < 1 year, 1–5 years or > 5 years. These workers used no personal protective equipment.

### Virological and serological analysis

Total nucleic acid was extracted from camel nasal swabs using EasyMag (Biomerieux, France) and screened for MERS-CoV RNA using a reverse-transcription qPCR (RT-qPCR) assay targeting the upstream elements of the Envelope (UpE) gene. Positive samples were confirmed by testing with a second RT-qPCR targeting the open reading frame 1a (ORF1a) gene [3,11].

Human sera were tested for MERS-CoV antibody using a MERS-CoV S1 spike enzyme-linked immunosorbent assay (ELISA; Euroimmun, Lübeck, Germany) according to manufacturer’s instructions and by a pseudoparticle neutralisation (ppNT) assay as described previously [12]. A ≥ 90% reduction of signal was considered as evidence of neutralisation in the ppNT assay.

## Results

Overall, 2,529 camels were tested, 38 of them being calves < 2 years, 1,400 aged 2–4 years and 1,091 aged > 4 years. None of the 1,300 camels tested from 12 October to 2 December 2015 were positive for MERS-CoV RNA. Those tested in the week of 11 January 2016 (n = 142) remained virus RNA negative. MERS–CoV was detected in subsequent weeks, five (2.6%) of 190 swabs in the week of 18 January, two (1%) of 199 in the week of 25 January, 12 (6.6%) of 183 in the week of 1 February, 16 (8.4%) of 190 in the week of 8 February, 12 (7.4%) of 162 in the week of 15 February and eight (5.2%) of 155 in the week of 22 February. None of eight tested in the week of 29 February were positive. Of the 2,529 camels tested, MERS-CoV RNA was detected in four (10.5%) of 38 aged < 2 years, 31 (2.2%) of 1,400 aged 2–4 years and 20 (1.8%) of 1,091 aged > 4 years.

A total of 311 abattoir workers were recruited for the serological study. Of these, 261 had occupational exposure to camels, with 243 workers having direct exposure to live camels, freshly killed camels or camel meat, and 18 having indirect exposure to camels. Fifty persons recruited in the study worked in the slaughterhouse with no occupational camel exposure. Many workers in the abattoir, including those without direct occupational exposure to camels, reported drinking fresh (unboiled) camel milk, drinking camel urine and the use of camel urine for medicinal purposes (Table). Irrespective of these modes of exposure, none of the 311 humans tested had any evidence of MERS-CoV antibody in their serum.

**Table:** Exposure to camels and history of camel product consumption in abattoir workers recruited for a Middle East respiratory syndrome coronavirus serological study, Kano, Nigeria, April–November 2016 (n = 311)

Characteristics	Exposure duration				Self-reported behavioural activities involving camel products^a^		
	< 1 year	1–5 years	> 5 years	Total^a^	Drinking camel milk	Drinking camel urine	Using camel urine as medicine
**Workers with exposure to camels in abattoir (n = 261)**							
Direct exposure (n = 243)							
Slaughtering	0	24	89	113	61/113	47/113	47/113
Butchering	0	6	10	16	7/16	4/16	4/16
Camel restraint/loading	0	5	11	16	15/16	8/16	8/16
Cleaning abattoir	0	4	16	20	15/20	12/20	12/20
Gutting camels	0	1	7	8	4/8	2/8	2/8
Separating meat from bone	0	1	1	2	0/2	0/2	0/2
Milking/urine collection	0	2	1	3	3/3	3/3	3/3
Blood collection	3	2	2	7	0/7	0/7	0/7
Meat collection/transport/storage	0	10	31	41	26/41	16/41	18/41
Marketing camels	0	8	52	60	29/60	16/60	16/60
Indirect exposure (n = 18)							
Roasted meat seller	0	0	1	1	1/1	0/1	0/1
Bone cracking^b^	0	5	13	18	10/18	9/18	9/18
Abattoir public service	0	0	1	1	0/1	0/1	0/1
**Workers with exposure to other animals in abattoir, but not camels (n = 50)**							
**Direct exposure**							
Slaughtering	0	1	0	1	1/1	1/1	1/1
Butchering	0	3	36	39	19/39	10/39	10/39
Animal restraint/loading	0	1	3	4	2/4	0/4	0/4
Cleaning abattoir	0	0	3	3	2/3	1/3	1/3
Gutting animals	0	0	1	1	1/1	1/1	1/1
Separating meat from bone	0	1	2	3	0/3	0/3	0/3
Meat collection/transport/storage	0	1	1	2	0/2	0/2	0/2
Marketing animals	0	0	4	4	2/4	0/4	0/4
Indirect exposure							
Bone cracking^b^	0	0	1	1	1/1	0/1	0/1

^a ^Some individuals have multiple exposures. Thus the numbers in the column(s) may add up to a number above 261 (for direct exposure) or 50 (for indirect exposure), respectively.

^b^ Meat is completely removed from the bone and the bone crushed into small pieces for food purposes, flavour and bone meal.

## Discussion

Camels (n = 132) in this abattoir in Kano had been previously studied in January 2015 and MERS-CoV RNA was detected in 11% of samples, while 96% were antibody positive [2]. In 2016 (this study), virus RNA detection in January–February ranged from 0–8.4% of camels sampled. The peak period of MERS-CoV activity in Kano appeared to be in February, about two months later than the peak of virus activity previously reported in Egypt [13].

The high rates of virus detection in camels during January–February in 2015 and 2016 suggest that workers in the abattoir had prolonged and intensive occupational exposure to MERS-CoV infected camels and camel carcasses, very likely, over many years, without the use of any personal protective equipment. In addition to occupational exposure to camels in the slaughtering process, of the 50 workers in the abattoir with no occupational exposure to camels or camel meat (Table) 15 reported frequent drinking of fresh camel milk, 10 drank fresh camel urine and 10 used camel urine as medicine. The complete absence of MERS-CoV antibodies in workers is striking. Although the serological assays were carried out using the spike protein of the prototype MERS-CoV strain EMC, we have shown that MERS-CoV from diverse parts of Africa, including Nigeria/NS004/2015, do not differ antigenically from the prototype MERS-CoV EMC strain [7]. Thus antigenic diversity is unlikely to explain the lack of seropositivity observed in camel-exposed individuals in Nigeria. The MERS-spike protein pseudoparticle neutralisation assay has comparable sensitivity to plaque neutralisation tests in detecting antibody in humans infected with MERS-CoV [14].

MERS-CoV seroprevalence in persons with occupational exposure to camels in the Arabian Peninsula was significantly higher than in the general population; five (3.6%) of 140 workers occupationally exposed to camels in Saudi Arabia investigated in 2013–14 and two of five camel slaughterers in a central animal market in Qatar tested in 2014 were MERS-CoV seropositive [8,15]. Two of 22 camel barn workers at a camel race track in Qatar were seropositive [15]. In our study, of 261 workers exposed to camels in the abattoir in Kano, Nigeria, none were seropositive. This seropositivity rate is significantly lower than that of the camel abattoir workers in Saudi Arabia (p = 0.0049, Fisher’s exact test) and that of the camel barn workers at a race track in Qatar (p = 0.0058). The finding of only negative test results in 113 camel slaughterers in this study yields a significantly different rate of seropositivity than that in camel slaughterers in an animal market in Qatar (p = 0.0014). Our results are concordant with that of a study in Kenya, East Africa, where there was no evidence of antibody in serum of 760 people with household or occupational exposure to MERS-CoV seropositive camels [9]. Another study of the general population from Kenya, found evidence of neutralising MERS-CoV antibody at very low antibody titre in two of 1,122 sera (0.18%) [10], comparable with a general population seroprevalence of 0.15% of sera from Saudi Arabia [8]. But it is unclear if these low antibody titres reflect actual infection with MERS-CoV. MERS-CoV from West Africa, including Nigeria, were genetically and phenotypically distinct from those in East Africa [7] and thus zoonotic potential of viruses from Nigeria may be different from those in Kenya. Overall, these data may suggest that the risk of MERS infection from exposure to infected camels may be lower in some African countries.

It should be noted that seroconversion is not invariable even in patients with MERS. In a cohort of patients with RT-PCR confirmed MERS in the South Korean outbreak in 2015 who were serologically followed up for one year, four of the six patients who had mild disease (i.e. did not require supplemental oxygen or mechanical ventilation) were negative by S1 ELISA, two were positive by plaque reduction neutralisation test (PRNT)90 (titre 1:10) and of these two only one was positive by ppNT (titre of 10) [16]. Although designated as having mild disease, with one exception, these patients, had chest infiltrates on X-ray, indicating lung parenchymal pathology. In another cohort of South Korean MERS patients, none of three persons with asymptomatic infection and only six of 10 patients with symptomatic disease without pneumonia seroconverted, whereas most patients with severe pneumonia did seroconvert [17]. Therefore it is possible that camel exposed individuals who get asymptomatic or mild infections may not seroconvert. Even in those who do develop detectable antibody, waning antibody titres may lead to negative serological results. Thus sero-epidemiological studies may well underestimate the true extent of MERS-CoV infection in humans. A recent study showed that virus-specific CD8 ^+^  T-cell responses were detected in mild or asymptomatic patients with MERS-CoV infection, even in the absence of serologic responses [18]. T-cell responses and their specificity for MERS-CoV should also be investigated in future studies for identifying evidence of zoonotic MERS-CoV infection in high risk groups.

In conclusion, we found no serological evidence of MERS-CoV infection in abattoir workers with extensive exposure to dromedaries with documented virus infection in winter months. lt is possible that MERS-CoV from West Africa may have lower zoonotic potential than current virus strains in the Arabian Peninsula [7]. Studying MERS-CoV in humans in Africa is an urgent priority. There is also a need for additional studies to genetically and phenotypically characterise MERS-CoV in Nigeria and other parts of Africa.
